# Features of infective native aortic aneurysms on computed tomography

**DOI:** 10.1186/s13244-021-01135-x

**Published:** 2022-01-08

**Authors:** Warissara Jutidamrongphan, Boonprasit Kritpracha, Karl Sörelius, Keerati Hongsakul, Ruedeekorn Suwannanon

**Affiliations:** 1 Department of Radiology, Faculty of Medicine, Prince of Songkla University, 15 Karnjanavanich Rd., Hat Yai, Songkhla 90110 Thailand; 2 Department of Surgery, Faculty of Medicine, Prince of Songkla University, 15 Karnjanavanich Rd, Hat Yai, Songkhla 90110 Thailand; 3 Department of Vascular Surgery, Rigshospitalet, Copenhagen, and Faculty of Health and Medical Sciences, University of Copenhagen, Copenhagen, Denmark

**Keywords:** Infective native aortic aneurysm, Aortic aneurysm, Computed tomography, Mycotic aortic aneurysm, Infected aortic aneurysm

## Abstract

**Background:**

Infective native aortic aneurysm (INAA) is a rare clinical diagnosis. The purpose of this study was to describe the CT findings of INAAs in detail.

**Methods:**

This was a retrospective single-center study of INAA patients at a major referral hospital between 2005 and 2020. All images were reviewed according to a protocol consisting of aneurysm features, periaortic findings, and associated surrounding structures.

**Results:**

One hundred and fourteen patients (mean age, 66 years [standard deviation, 11 years]; 91 men) with 132 aneurysms were included. The most common locations were infrarenal (50.8%), aortoiliac (15.2%), and juxtarenal (12.9%). The mean transaxial diameter was 6.2 cm. Most INAAs were saccular (87.9%) and multilobulated (91.7%). Calcified aortic plaque was present in 93.2% and within the aneurysm in 51.5%. INAA instability was classified as contained rupture (27.3%), impending rupture (26.5%), and free rupture (3.8%). Rapid expansion was demonstrated in 13 of 14 (92.9%) aneurysms with sequential CT studies. Periaortic inflammation was demonstrated as periaortic enhancement (94.7%), fat stranding (93.9%), soft-tissue mass (92.4%), and lymphadenopathy (62.1%). Surrounding involvement included psoas muscle (17.8%), spondylitis (11.4%), and perinephric region (2.8%). Twelve patients demonstrated thoracic and abdominal INAA complications: fistulas to the esophagus (20%), bronchus (16%), bowel (1.9%), and inferior vena cava (IVC) (0.9%).

**Conclusion:**

The most common CT features of INAA were saccular aneurysm, multilobulation, and calcified plaques. The most frequent periaortic findings were enhancement, fat stranding, and soft-tissue mass. Surrounding involvement, including psoas muscle, IVC, gastrointestinal tract, and bronchi, was infrequent but may develop as critical INAA complications.

## Key points

- The characteristic INAA features were multilobulated saccular aneurysm with periaortic abnormality.
- Presence of calcified atherosclerotic plaque made INAA indistinguishable from degenerative aneurysm.
- Psoas muscle involvement and fistulas were rare but may represent critical complications.

## Introduction

Infective native aortic aneurysm (INAA), also known as mycotic aortic aneurysm, is a challenging disease in respect of making the diagnosis [1, 2]. It is a rare entity found in only 0.6‒2.6% of all aortic aneurysms in western countries and up to 13% in Asia [3, 4]. INAA may develop through various pathophysiological ways according to the amended Wilson's classification [5, 6]: (1) due to septic emboli lodging in the aortic wall from infective endocarditis; (2) blood-borne bacteria inoculated in the aortic wall during bacteremia; (3) infection of a pre-existing aneurysm due to blood-borne bacteria; and (4) aneurysms developing in patients with advanced human immunodeficiency virus infection.

Due to INAA variability and non-specific manifestations, a delayed or missed diagnosis may lead to a fatal outcome. Therefore, making an accurate and timely diagnosis is crucial for proper management, including securement of the microbiological specimen, initiation of appropriate antibiotic therapy, and surgery.

The European Society for Vascular Surgery 2019 guidelines [7] on abdominal aortic aneurysms recommend that the diagnostic workup of INAA should consist of a combination of: (1) clinical presentation; (2) laboratory results; and (3) computed tomography (CT) findings. Since there is no pathognomonic symptom or test for the disease, CT is thus the cornerstone for the diagnosis of INAA. Typical CT findings have been reported to consist of saccular shape, multilobulated or eccentric aneurysm, periaortic gas, soft-tissue mass, rapid expansion (days) or rupture or both, atypical location (e.g., para-visceral), and multiple aneurysms in different areas [7, 8].

However, several causes of aortitis might also mimic INAA identification on CT images [9]. Moreover, detailed studies on CT findings of INAA are scarce and small partly due to the rarity of the disease and partly due to the fact that CT findings have been reported mainly as a subordinate finding in studies reporting treatment outcome [2].

This study aimed to describe the features of INAA on CT at presentation, including details of the aneurysm, presence of aortic wall calcification, and periaortic findings, as well as associated findings in other organs.

## Materials and methods

This is a retrospective study of patients treated for INAA at a major referral center between September 2005 and April 2020. Patients were identified from the internal hospital registry in the vascular surgery and radiology databases. The institutional review board of the Faculty of Medicine, Prince of Songkla University approved the study. Informed consent was not required due to the retrospective nature of the study. The CT examinations were performed on a 64-slice multidetector computed tomography (MDCT) scanner from 2015 to 2018, a 160-slice MDCT scanner from 2015, and a 512-slice MDCT scanner from 2018.

The diagnostic workup was performed according to the newly proposed algorithm in the European Journal of Vascular and Endovascular Surgery [7]. The diagnostic algorithm was a combination of three clinical criteria: (1) clinical presentation; (2) laboratory results; and (3) CT imaging. All patients in this study had to meet all three of the criteria for a definite diagnosis to be included in this study. Patients with a history of previous aortic surgery or an incomplete/inadequate initial CT study were excluded.

The retrospective review of all patients included demographic data on patient characteristics (i.e., sex, age, medical history including immunodeficiency and comorbidities, treatments causing relative immunodeficiency, symptoms [pain, fever, shock], concurrent infection), laboratory and microbiological findings (inflammatory markers: C-reactive protein, levels of leukocytes, cultures from blood/tissues, results of a polymerase chain reaction assay for bacterial identification), and detailed CT findings.

### CT analysis

All CT examinations were reviewed by a senior consultant in cardiovascular radiology with 8 years of experience and an in-training resident with 3 years of experience at the same time to make the consensus. Despite being aware that all images were of INAA, the reviewers were blinded to the clinical information of each patient.

The CT examinations were evaluated on three categories. First were the features of the aneurysm that included the number of aneurysms (single or multiple), location, size (measured in maximum transaxial diameter (cm) of outer wall to outer wall of aneurysm including periaortic soft tissue), rupture status (divided into no rupture, impending rupture, contained rupture, free rupture), rapid progression, shape (saccular or fusiform), contour (multilobulated or non-lobulated), and the presence of calcified atherosclerotic plaque. The extent of calcification was classified into five types based on the findings in this study: type 0 saccular aneurysm without calcification; type 1 saccular aneurysm with calcified aneurysm neck; type 2 saccular aneurysm with calcification of the aorta more than 1 cm from the aneurysm; type 3 saccular aneurysm with calcification within the aneurysm wall; type 4 fusiform aneurysm with calcified intima; and type 5 fusiform aneurysm with peripheral calcification. Second were the periaortic findings that included aneurysmal wall enhancement, fat stranding, soft-tissue mass, lymphadenopathy, fluid, and the presence of ectopic gas. Third was the associated surrounding organ involvement according to either the overall or specific location of the INAA (e.g., abdominal aorta and thoracic aorta), such as psoas muscle, perinephric region, aortocaval fistula, the hollow viscus (bowel, bronchus, esophagus), and spondylitis.

### Statistical analysis

The sample size was calculated by the infinite population proportion formula, which revealed at least 100 subjects to represent justification of the sample size. All recorded data were assessed for normality with histograms. Continuous data are expressed as mean and standard deviation (SD) and categorical variables as proportions (%). Non-normally distributed continuous data are expressed as median and range. All statistical analyses were performed by a statistician using R software, version 4.0.3 (https://www.r-project.org/).

## Results

### Patient characteristics

**Fig. 1:**
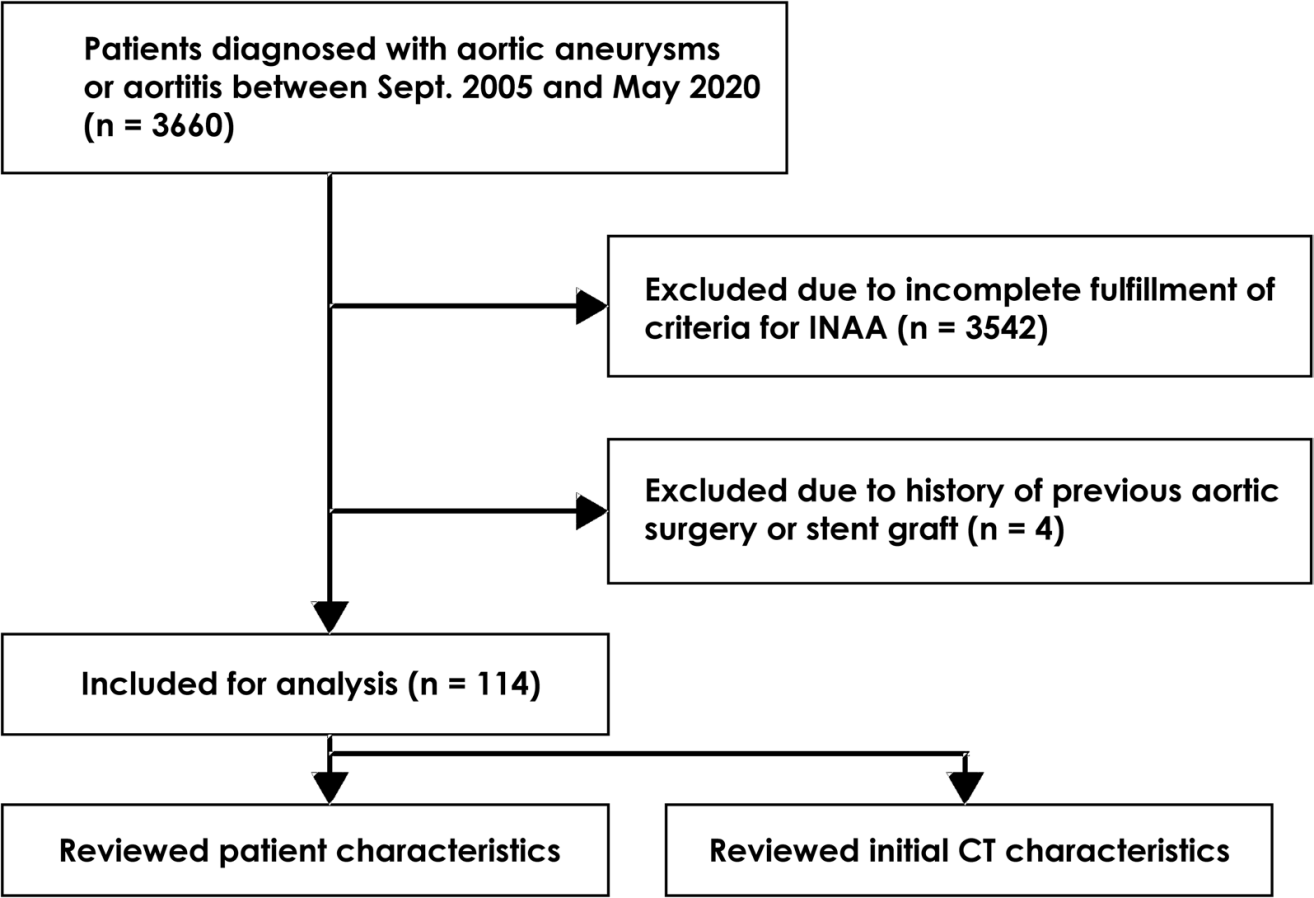
Flow diagram of this study

During the study period, 3660 patients were either diagnosed with aortic aneurysms (*n* = 3537) or aortitis (*n* = 123). Among these patients, 3546 patients were excluded, leaving 114 patients who fulfilled all criteria for INAA. The study flow diagram is shown in Fig. 1. The majority of the patients were male (91/114; 79.8%) and the mean (SD) age was 66 (11) years (range 41‒92 years). The patient characteristics are provided in Table 1. Twenty-nine (25.4%) patients had at least one concomitant infection, documented as urinary tract infection (*n* = 13), septic arthritis (*n* = 7), abscess (*n* = 5), pulmonary tuberculosis (*n* = 4), enterocolitis (*n* = 2), and osteomyelitis (*n* = 1). Forty (35.1%) cases had positive blood cultures. Almost half of the patients (47/114; 41.2%) had antibiotic administration prior to obtaining the blood cultures at this major hospital, and only a small number (7/47; 14.9%) of them had positive culture results. Since most of the patients (99/114; 86.8%) were referred from surrounding primary hospitals, more than one-third (39/99; 39.4%) had documented previous antibiotic therapy, while such data were unavailable in approximately half of them (45/99; 45.5%).

**Table 1:** Characteristics in INAA patients (*n* = 114)

Variable	*n* (%)
Age (year)^a^	66 (11)
Male	91 (79.8)
*Medical history*	
Hypertension	58 (50.9)
Diabetes mellitus	40 (35.1)
Ischemic heart disease	10 (8.8)
Renal failure	10 (8.8)
HIV infection	6 (5.3)
Cerebrovascular disease	5 (4.4)
COPD	3 (2.6)
Steroid treatment	3 (2.6)
*Clinical manifestation*	
Abdominal pain	86 (75.4)
Back pain	21 (18.4)
Fever	95 (83.3)
Circulatory shock	10 (8.8)
Hemoptysis	5 (4.4)
UGIB	6 (5.3)
LGIB	3 (2.6)
Urinary tract infection	13 (11.4)
Septic arthritis	7 (6.1)
Abscess	5 (4.4)
Pulmonary tuberculosis	4 (3.5)
Enterocolitis	2 (1.8)
Osteomyelitis	1 (0.9)
*Laboratory data*	
Leukocytosis	107 (93.9)
Elevated ESR	59 (51.8)
Elevated CRP	59 (51.8)
Positive blood culture	40 (35.1)
Salmonella	15 (13.2)
Staphylococcus	8 (7)
Streptococcus	6 (5.3)
Melioidosis	5 (4.4)
Enterococcus coli	5 (4.4)
Mycobacterium	2 (1.8)
Other	1 (0.9)

*INAA* infective native aortic aneurysm, *HIV* human immunodeficiency virus, *COPD* congestive obstructive pulmonary disease, *UGIB* upper gastrointestinal bleeding, *LGIB* lower gastrointestinal bleeding, *ESR* erythrocyte sedimentation rate, *CRP* C-reactive protein

^a^Data are mean with standard deviation in parenthesis

### Features of infective native aortic aneurysm on CT

**Fig. 2:**
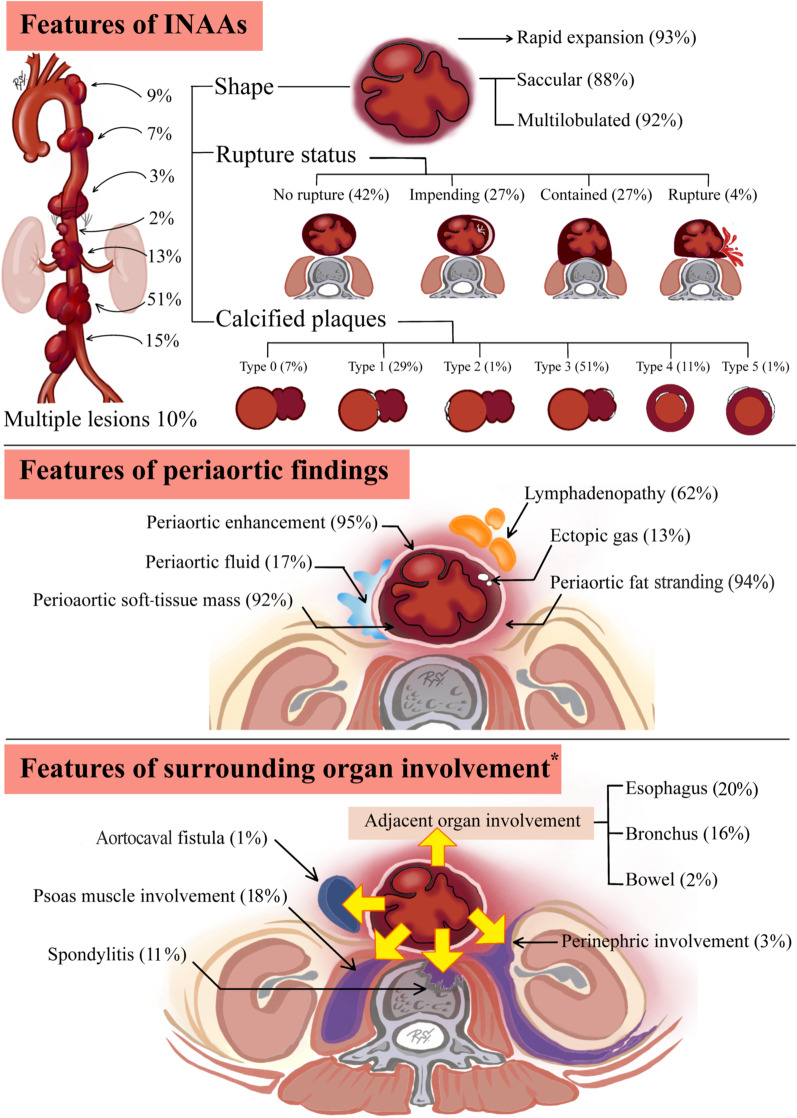
Summary of all CT findings in INAA in 132 aneurysms in three catagories: (1) features of infected aneurysm; (2) periaortic findings; and (3) surrounding organ involvement. Asterisk (*) indicates surrounding organ involvement according to the anatomical structure and location of INAA

The CT findings were divided into three categories: (1) features of the infected aneurysm; (2) periaortic tissues; and (3) associated structures (Fig. 2).

#### Features of the infective aneurysm

The distributions of INAA were in the aortic arch (12/132; 9.1%), descending aorta (9/132; 6.8%), thoracoabdominal aorta (4/132; 3%), suprarenal aorta (3/132; 2.3%), juxtarenal aorta (17/132; 12.9%), infrarenal aorta in half of the patients (67/132; 50.8%), and aortoiliac location (20/132; 15.2%). In 12 (10.5%) patients, multiple aneurysms were found (Table 2).

**Table 2:** Background and features of 132 aneurysms on initial CT in 114 patients with INAA

Patients	*n* = 114
Single aneurysm	102 (89.5)
Multiple aneurysms	12 (10.5)
CT findings of aneurysms (*n* = 132)	*n* (%)
*Location*	
Arch	12 (9.1)
Descending aorta	9 (6.8)
Thoracoabdominal	4 (3)
Suprarenal	3 (2.3)
Juxtarenal	17 (12.9)
Infrarenal	67 (50.8)
Aortoiliac	20 (15.2)
Size (cm)^a^	6.2 (2.3)
*Rupture status*	
No rupture	56 (42.4)
Impending rupture	35 (26.5)
Contained rupture	36 (27.3)
Rupture	5 (3.8)
*Shape*	
Saccular	116 (87.9)
Fusiform	16 (12.1)
Multilobulated contour	121 (91.7)
Rapid expansion^b^	13 (92.9)
*Calcified atherosclerotic plaque*	
Type 0	9 (6.8)
Type 1	38 (28.8)
Type 2	1 (0.8)
Type 3	68 (51.5)
Type 4	14 (10.6)
Type 5	2 (1.5)

*INAA* Infective native aortic aneurysm

^a^Data are mean with standard deviation in parenthesis

^b^Data represent 13 of 14 patients with sequential CT studies

**Fig. 3:**
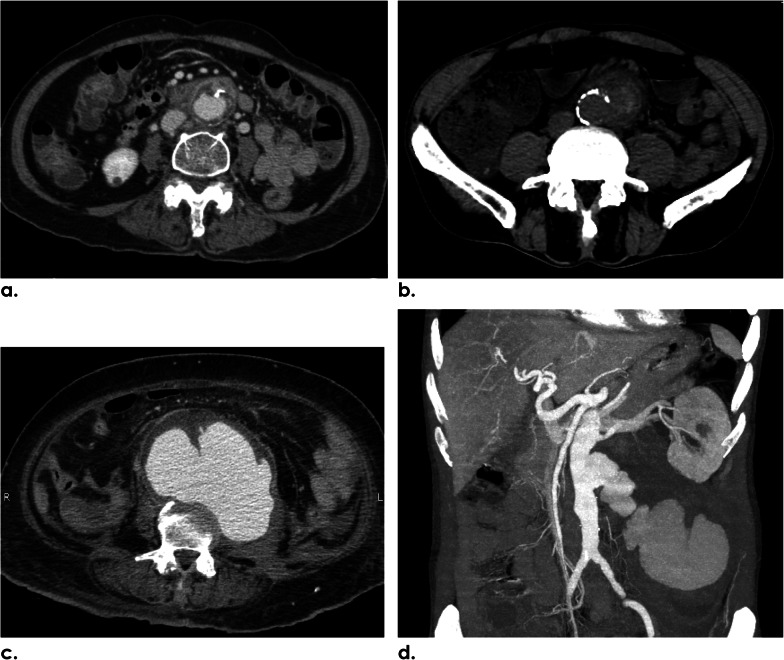
CT findings of INAA instability. **a** Axial CECT image of a 77-year-old female patient reveals an infrarenal aneurysm and periaortic soft-tissue mass without signs of instability. **b** Axial NECT image of a 68-year-old male patient depicts hyperattenuation of the peripheral thrombus in an infrarenal aneurysm indicating impending rupture. **c** Axial CECT of a 61-year-old female patient demonstrates a multilobulated infrarenal aneurysm with its posterior wall draping along the anterior vertebral body, draped aorta sign, representing contained rupture. **d** Coronal MIP angiographic CT image of a 63-year-old male patient reveals active contrast extravasation from the infrarenal INAA into the retroperitoneal cavity indicating free rupture. *INAA* infective native aortic aneurysm, *CECT* contrast-enhanced computer tomography, *NECT* non-enhanced computed tomography, *MIP* maximum intensity projection

**Fig. 4:**
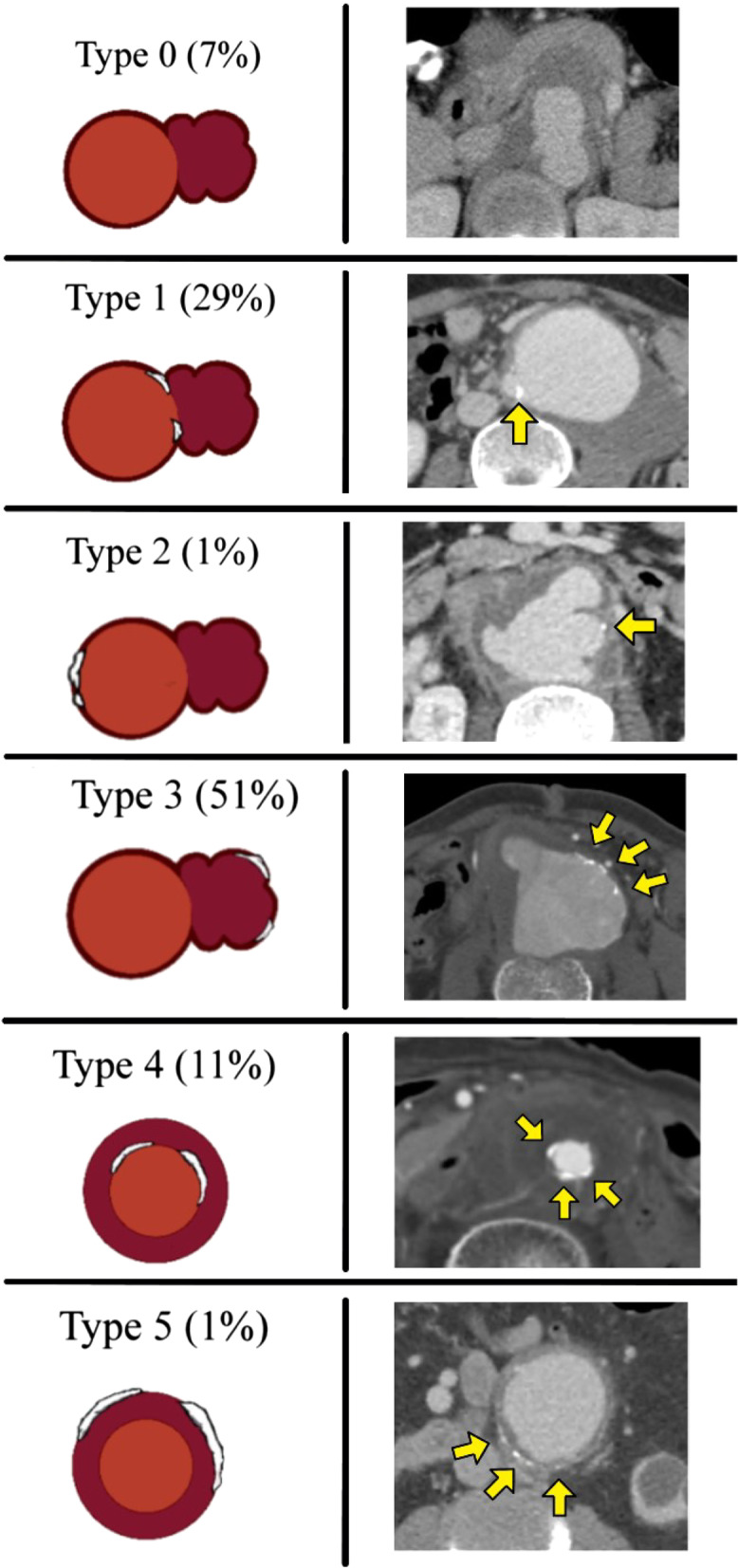
Anatomical classification of the INAA with associated calcified aortic plaques: type 0 saccular aneurysm without calcification; type 1 saccular aneurysm with calcified aneurysm neck; type 2 saccular aneurysm with aortic calcification far from aneurysm (more than 1 cm); type 3 saccular aneurysm with calcification within aneurysm wall; type 4 fusiform aneurysm with calcified intima; and type 5 fusiform aneurysm with peripheral calcification. Yellow arrows indicate calcified plaque on CT

Of the 132 aneurysms, the types of aneurysms found were saccular (116/132; 87.9%) and fusiform (16/132; 12.1%), while most of them showed a multilobulated contour (121/132; 91.7%). The mean (SD) aneurysmal sac size measured in the maximal transaxial diameter was 6.2 cm (2.3 cm). In 56 (42.4%) of the 132 aneurysms, no sign of rupture (Fig. 3a) was identified. The CT findings of aneurysm instability in this study were impending rupture (Fig. 3b) in 35 (26.5%) aneurysms, contained rupture (Fig. 3c) in 36 (27.3%) aneurysms, and free rupture (Fig. 3d) in 5 (3.8%) aneurysms. In the patients with sequential CT studies performed, the median values of rapid expansion in size and time of the aneurysms were 1.1 cm (range 0.3–4 cm) and 69 days (range 5–136 days), respectively, found in 13 of 14 patients (92.9%). Almost all INAA (123/132; 93.2%) in this study demonstrated associated calcified aortic plaques that were classified into five types (Table 2). The most common type found in half of the patients was type 3 saccular aneurysm with calcification within the aneurysm wall (68/132; 51.5%) while the second and third most common types were type 1 saccular aneurysm with calcified aneurysm neck (38/132; 28.8%) and type 4 fusiform aneurysm with calcified intima (14/132; 10.6%), respectively (Fig. 4).

#### Features of the periaortic tissues on CT

**Fig. 5:**
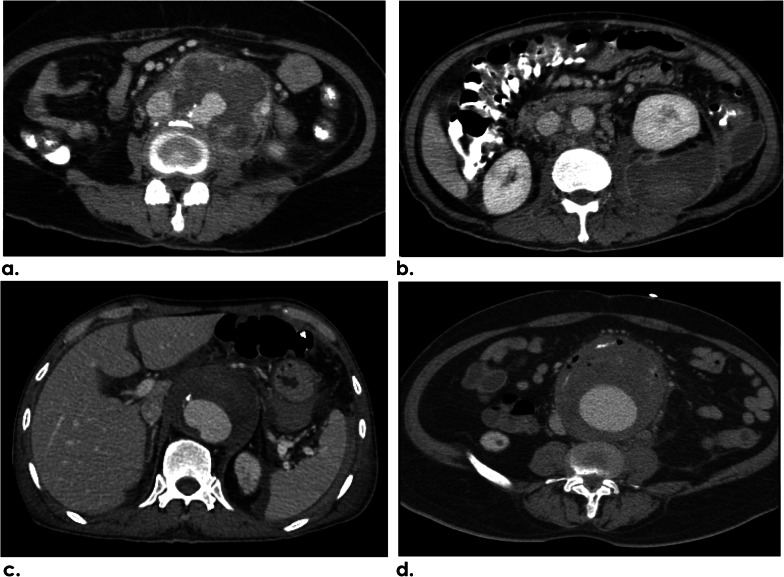
Periaortic CT findings of INAA. **a** Axial CECT image of a 58-year-old female patient demonstrates enhancing periaortic soft-tissue mass and fat stranding surrounding the multilobulated infrarenal aneurysm. **b** Axial CECT image of a 69-year-old male patient reveals a group of periaortic lymph nodes and fat stranding adjacent to the level of an infrarenal aneurysm. Left perinephric space involvement of INAA is also evident. **c** Axial CECT image of a 57-year-old female patient reveals a suprarenal aneurysm with periaortic soft-tissue mass and surrounding hypodense fluid extending to the peritoneal and retroperitoneal spaces. **d** Axial CECT image of a 62-year-old male patient shows periaortic soft-tissue mass with a few ectopic gas bubbles in the infrarenal aneurysm. *INAA* infective native aortic aneurysm, *CECT* contrast-enhanced computer tomography

Periaortic enhancement (125/132; 94.7%), fat stranding (124/132; 93.9%), and soft-tissue mass (122/132; 92.4%) were the most common periaortic findings (Table 3 and Fig. 5a). About two-thirds of the patients (82/132; 62.1%) demonstrated periaortic lymphadenopathy (Fig. 5b). The less frequent features were periaortic fluid (22/132; 16.7%) and ectopic gas (17/132; 12.9%) (Fig. 5c, d).

**Table 3:** Periaortic findings of aneurysms on initial CT in INAA patients

CT findings of aneurysms (*n* = 132)	*n* (%)
Periaortic enhancement	125 (94.7)
Periaortic fat stranding	124 (93.9)
Periaortic soft-tissue mass	122 (92.4)
Periaortic lymphadenopathy	82 (62.1)
Periaortic fluid	22 (16.7)
Presence of ectopic gas	17 (12.9)

Data are presented as number (%)

*INAA* infective native aortic aneurysm

#### Features demonstrated in the surrounding organs on CT

**Fig. 6:**
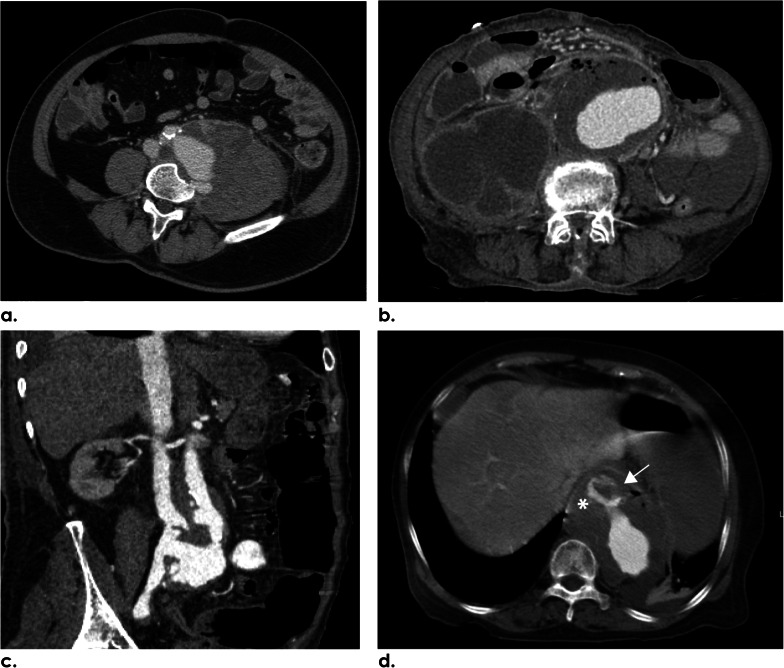
Surrounding organ involvement of INAA. **a** Axial CECT of a 58-year-old male patient depicts an infrarenal aneurysm with enhancing periaortic soft-tissue mass involving the left psoas muscle. The concave eroded anterior border of the adjacent vertebral body represents spondylitis. **b** Axial CECT of an 84-year-old female patient presenting with gastrointestinal hemorrhage reveals multiple air bubbles in the infrarenal aneurysm connecting to the adjacent duodenum, suggestive of aortoenteric fistula. A rim-enhancing fluid collection involving enlarged right psoas muscle is also noted. **c** Coronal angiographic CT image of a 66-year-old male patient reveals early enhancement of the IVC connecting to the aorta via multilobulated aortoiliac aneurysm, suggestive of aortocaval fistula. **d** Axial CECT of an 84-year-old female patient reveals active contrast extravasation (white arrow) from INAA into the esophagus. Asterisk (*) indicates aortoesophageal fistula. *INAA* infective native aortic aneurysm, *CECT* contrast-enhanced computer tomography, *IVC* inferior vena cava

Overall, the INAAs were classified according to anatomical location as thoracic aortic aneurysms (25/132; 18.9%) and abdominal aortic aneurysms (107/132; 81.1%). In the intra-abdominal region, the two most common surrounding structure involvements were psoas muscle (19/107; 17.8%) and perinephric region (3/107; 2.8%) (Table 4, Fig. 6a, b). Aortocaval fistula was rarely found (1/107; 0.9%) (Fig. 6c). Hollow viscus involvement, such as the bowel (2/107; 1.9%) (Fig. 6b), was scarce in the abdomen but appeared relatively more prevalent in the thorax as suspected esophageal (Fig. 6d) and bronchial complications, up to 20% (5/25) and 16% (4/25), respectively. Spondylitis was the surrounding structural involvement regardless of the INAA location found in 11.4% (15/132) (Fig. 6a).

**Table 4:** Features associated with the surrounding structures of 132 aneurysms on initial CT of 114 patients with INAA

Location of INAA	Abdominal aortic aneurysm (*n* = 107)	Thoracic aortic aneurysm (*n* = 25)
*CT findings*		
Psoas involvement	19 (17.8)	–
Perinephric involvement	3 (2.8)	–
Aortocaval fistula	1 (0.9)	–
*Hollow viscus involvement*		
Bowel	2 (1.9)	–
Bronchus	–	4 (16)
Esophagus	–	5 (20)
Spondylitis	15 (11.4)	

Data are presented as number (%)

*INAA* infective native aortic aneurysm

## Discussion

This is the largest study on INAA patients to report detailed findings using CT [1, 10]. The main findings of this study were that the majority of patients had saccular aneurysms often with a multilobulated contour and an infrarenal aortic location. Also, as opposed to prior conception, many of these aneurysms contained atherosclerotic plaques.

Although calcification at the aneurysmal wall is commonly found in degenerative aortic aneurysms, it has been rarely reported in INAA, which seems to be an errant misconception [1, 10]. Several pathological studies of INAA have shown acute transmural inflammation superimposed on the atherosclerotic aorta as the typical pathogenesis of INAA [11, 12], thereby supporting calcified plaque as its common CT feature. Therefore, the presence of calcified plaque could not distinguish INAA apart from a degenerative aortic aneurysm [1]. Moreover, the infrarenal aorta has been the most common site of both INAA and degenerative aortic aneurysm, inferring that INAA is a disease commonly affecting elderly patients with aged aortas and comorbidities causing atherosclerosis [2, 6, 13].

The inflammation process involving the compromised aortic wall would also cause an aggressive appearance of the aneurysm including the saccular shape, multilobulated contour, large initial aneurysm sac, and rapid progression in size as the primary CT features of INAA [1, 10, 14, 15]. Furthermore, evidence of aneurysm instability (i.e., impending rupture, contained rupture, or free rupture) from the CT findings was also found in more than half of the patients with INAA.

The typical periaortic characteristics of INAA in this study were soft-tissue mass, enhancement, fluid, and fat stranding, which were likely secondary to the infection and subsequent inflammation [1, 8, 10, 16–18].

Although some CT findings, including periaortic fluid and ectopic gas, were previously described as uncommon [1, 8, 10, 19–21], periaortic lymphadenopathy, which supported the diagnosis of INAA, was found in two-thirds of the patients in this study. However, periaortic lymphadenopathy was reported in only a few previous studies [14, 17]. Since lymphadenopathy has been one of the characteristics to indicate surrounding active infection/inflammation, its role in determining the activity of infection and the treatment response of INAA could be another opportunity for future researchers [22].

The features associated with the surrounding structures were less often noticed. However, they could be found as INAA culminated in secondary complications and thus were noteworthy for urgent management. The psoas muscle could either have been the primary site of abscess or hematoma secondary to a ruptured INAA [1, 23, 24]. These etiologies can be indistinguishable on CT, which leaves percutaneous aspiration as the most appropriate means to simultaneously treat and secure a specimen for culture and polymerase chain reaction analysis and to differentiate the two conditions from each other [25, 26].

Despite the rare incidence, ectopic gas should raise suspicion of a gastrointestinal structure or bronchus involvement apart from being evidence of infection [18, 23, 27]. However, contrast extravasation from INAA into a hollow viscus was rarely seen on CT in this study. Having said that, a definite diagnosis of aortic fistula prior to exsanguination is only possible from CT findings when appropriate clinical information, such as herald bleeding, is present [28, 29]. Also, thoracic INAA is relatively infrequent compared to abdominal INAA [30, 31]. On the contrary, there is a greater proportion of hollow viscus complications from esophagus and bronchus involvement in thoracic INAA than bowel involvement in abdominal INAA in this study. Spondylitis and perinephric involvement were also uncommon concomitants, as reported in previous studies [1, 10, 23, 32, 33]. Only a few previous cases of aortocaval fistula in the acute setting of INAA have been reported [10, 23].

Our study had some limitations. Even though this is the largest study of CT characteristics of INAA, the number of cases is relatively small due to the rare prevalence. The high rates of previous antibiotic treatment before obtaining blood culture were responsible for the low rate of positive blood culture results in this study. However, the rate of negative cultures in this study was in line with the literature [2, 34]. Maximal diameter from the outer border to the outer border of the aneurysm on axial CT image was the standard measurement method in this study. Such simplicity led to a good comparison to determine treatment response in the follow-up examinations. However, to date no standard measurement of INAA sac size has been proposed [16, 35].

In conclusion, infective native aortic aneurysms most commonly occur in the infrarenal aorta and are usually associated with calcified atherosclerotic plaques. The most common aneurysmal features are saccular shape, multilobulated contour, aneurysmal instability, and rapid expansion. The typical periaortic findings are enhancement, fat stranding, and soft-tissue mass. Involvement of the adjacent psoas muscle, gastrointestinal structures, and bronchi are infrequent but do occur as critical complications to INAA.

## Data Availability

The data that support the findings of this study are available on request from the corresponding author, (RS). The data are not publicly available due to restrictions, e.g., they contain information that could compromise the privacy of research participants.

## Abbreviations

CT: Computed tomography
INAA: Infective native aortic aneurysm
IVC: Inferior vena cava
MDCT: Multidetector computed tomography
SD: Standard deviation
